# Population health intervention research training: the value of public health internships and mentorship

**DOI:** 10.1186/s40985-018-0084-9

**Published:** 2018-04-02

**Authors:** Anne-Marie Hamelin, Gilles Paradis

**Affiliations:** Department of Epidemiology, Biostatistics and Occupational Health, McGill University, Montreal, Quebec Canada

**Keywords:** Population health intervention research, Internships, Mentorship, Competency-based research training, Capacity building, University-public health partnership

## Abstract

**Background:**

Better alignment between academia and public health practice and policies are critical to improve public health actions. Training of future researchers to address complex issues and to conduct transdisciplinary and collaborative research will help improve this alignment. In this paper, we describe the role of internship placements and mentorship for trainees’ skills development in population health intervention research and the benefits of embedding research trainees within public health organizations.

**Methods:**

This qualitative descriptive study assessed the perceptions of the role and benefits of internships and mentorship for population health intervention research training among former doctoral and postdoctoral students, public health mentors, and senior public health managers who participated in the 4P Program, a research training program which bridges academic training and the public health system in Quebec, Canada. Two types of interviews were conducted: telephone semi-structured interviews by an external evaluator and face-to-face trainee “exit” interviews by the Program co-director. Semi-annual evaluation reports from each trainee were also reviewed. Qualitative data were subjected to a thematic analysis.

**Results:**

Internships provided trainees with a working knowledge of the public health system and the context in which decisions and public health interventions are implemented. It was an opportunity for trainees to interact with knowledge-user partners and assess the gap between research and practice. Effective mentorship was key to help trainees interpret the public health reality and develop population health intervention research skills. Trainees learned to ask the “how” questions that are critical for in-depth understanding of complex interventions and the conditions under which they can be best implemented. Conditions of success of internships and mentorship for population health intervention research included the alignment of the interests between the trainee, the mentor and the public health organization, quality mentoring, and the acquisition of specific population health intervention skills, especially collaborative research skills.

**Conclusions:**

The findings suggest that public health internships and mentorship facilitate trainee engagement in applied public health research.

**Electronic supplementary material:**

The online version of this article (10.1186/s40985-018-0084-9) contains supplementary material, which is available to authorized users.

## Background

Increasing research capacity in population health intervention research (PHIR) requires better alignment between academia and public health practice [1–3] and a greater ability of researchers to conduct transdisciplinary and collaborative research [4–9]. Internships and mentorship are perceived to be important aspects of training and career development for researchers [10–18]. However, whether and how they enhance research training in public health is poorly understood.

Our objectives are to examine (1) the role of internship placements and mentorship for trainees’ skills development in PHIR and (2) the benefits of embedding research trainees within public health organizations (PHOs), based on the perspective of trainees and mentors who participated in the 4P Program, a university-public health partnership research training program [19].

### Program description

The main objective of the 4P Program [19] was to prepare doctoral and post-doctoral students from Quebec universities to conduct applied research on population and public health interventions (PHI) in PHOs. Over 12 years (2003–2015), 63 doctoral and post-doctoral trainees were funded to participate in the Program. It offered training based on core competencies development, including (1) foundational knowledge of public health, (2) transdisciplinary research, (3) intervention research ethics, (4) development of research networks and partnerships, (5) knowledge translation, and (6) research career management.

A unique component of this Program involved embedding trainees into a PHO under the supervision of a public health mentor. Doctoral candidates were required to spend 60% of their time for 3 years in a PHO and post-doctoral fellows, 80% of their time for 2 years. Internship objectives were to (1) provide a public health field experience to trainees; (2) increase their understanding of the public health system, its values and its needs; (3) understand how public health problems are conceptualized, researched, and solved in PHOs; and (4) develop cross-disciplinary working relationships with practitioners while appreciating the challenges of collaborative work and knowledge translation in implementing evidence-informed interventions.

Ten PHOs hosted trainees. A total of 37 departments from seven universities and 31 disciplines were represented among trainees and mentors. Sixty percent of mentors who supervised trainees were employed by PHOs, 47% had a PhD, 44% were MDs, and 38% mentored more than one 4P trainee. Mentorship took place through regular in-person meetings. The roles and responsibilities of mentors are presented in Table 1. Each trainee was expected to play an important role in the activities of the mentor’s public health team, while maintaining progress in their graduate studies. The placements’ specific experiences and topics of study were varied and reflected current public health priorities.

**Table 1:** Roles and responsibilities of the 4P Program mentors

Roles	Responsibilities
Support and encourage trainee’s development and supervise her/his internship in the public health organization (PHO)	Meet with the trainee at regular intervals to review progress and report on this progress twice a year to the 4P Program Director
	Facilitate the material and intellectual integration of the trainee in the PHO in accordance with the objectives of the 4P Program
	Ensure cohesion of the expectations of the mentor, trainee, and the 4P Program
	Participate, as appropriate, in the 4P Program seminar series. The mentor is asked to commit to participating in at least one seminar per term
Mentor the trainee	Share knowledge, experience, and lessons learned
	Critically examine fundamental issues in public health
	Discuss public health research issues
	Provide the trainee with stimulating opportunities and spaces for reflection, research, and intervention that will contribute to the development of:
	• Professional skills (e.g., ethics, adoption of a public health perspective, effective management of research programs and projects, interdisciplinary teamwork—interactions within a research team and also with professionals, decision-makers, and community members—leadership, development and maintenance of a network of collaborators, research budget preparation). • Methodological skills (e.g., selecting and applying appropriate intervention research methods, use of new information technologies) • Writing skills (e.g., writing research grants, scientific and lay writing) • Knowledge translation skills (e.g., process of collaboration with knowledge-user partners, dissemination, and application of knowledge to support public health interventions)
	Facilitate collaborative research
	Listen to and offer sufficiently frequent and regular feedback
Encourage research career development	Help the trainee develop skills in research career management and planning
	Offer support to the trainee in his professional decisions

## Methods

**Fig. 1:**
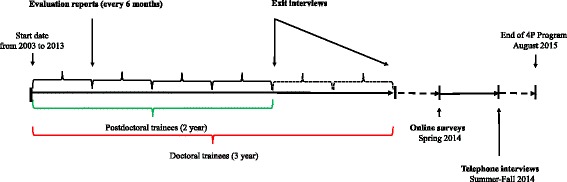
Timeline of different evaluation components of the 4P Program

The Program’s overall evaluation has been described [19]. For the current qualitative study, we assessed the perceptions of Program participants on the role and benefits of internships and mentorship for PHIR training through multiple means (Table 2; see also a timeline of the evaluation in Fig. 1). Telephone semi-structured interviews (TI) were conducted by an external evaluator with former trainees, mentors, and senior public health managers to assess their perceptions of the Program’s contributions to trainee outcomes. For these interviews, 18 out of a total of 44 trainees who had completed the Program by January 2014 were randomly selected, ensuring balance across PHOs (local, regional, national level), disciplinary field of origin (social sciences and humanities, natural and life sciences, other), and research areas (prevention, health promotion, organization of health care and services, public policies). Six public health mentors (out of ten sampled) and two senior PHO managers (out of five sampled) were also interviewed. Interviews were recorded and summary notes transcribed for thematic analyses. From 2009 onward, trainees had face-to-face, end-of-Program “exit interviews” (EI) that lasted between 90 and 120 min with the Program co-director to assess the attainment of learning objectives and perceived impact of the Program on their training. Thematic analyses were conducted from detailed notes from 33 exit interviews (out of 37). In addition, the semiannual trainee self-reported internship evaluations (SAER, 2003–2014) were reviewed. Finally, online surveys completed by 38 out of a total of 63 former trainees and 41 out of a total of 55 mentors provided additional information for the acquisition of skills in PHIR and were used to add to the triangulation of methods and cross verify the convergence of the results.

**Table 2:** Data collection methods and sources

Methods and sources^a^	Data collected
Telephone interviews (with an external consultant) (TI) (1, 2, 3)	Nature, usefulness, and extent of the contribution of internships and mentorship to (a) current research practices, research orientations, and career development (trainees only) and (b) trainees PHIR skills acquisition (mentors only)
	Program adaptation to the PHO needs and most important contributions of the Program (managers only); facilitators and barriers to PHIR training; recommendations for Program improvement
Exit interviews (with the Program co-director) (EI) (1)	Achievement of the trainee personal learning objectives (career development, professional network development, transdisciplinary intervention research, knowledge translation, public health concepts and systems knowledge, ethics) and the contribution made by the internships and mentorship
Semiannual evaluation reports (SAER) (1, 2)	Frequency of encounters between trainees and mentors and comments from trainees and mentors on the progress towards achieving personal learning objectives, satisfaction with the mentoring relationship, and suggested improvements to any aspect of the Program
Online surveys (1, 2)	Contribution of internships and mentorship to enhance: a broad vision of public health interventions, an openness towards other disciplines, an understanding of ethics
	Trainees only: skills for interacting and working with people from other disciplines, creating research partnerships, sharing knowledge

^a^Sources: 1 trainees, 2 mentors, 3 senior PHO managers

Additional file 1

We examined the role of internships and mentorship based on the unique features of PHIR [20] in which PHIs are conceived as complex systems in continuous interaction within a changing environment [21]. PHIR questions of interest emphasize the “how” and revolve around the relevance, coherence, responsiveness, achievements, and impact of interventions [20]. We therefore paid attention to the broad knowledge and particular competencies needed by researchers to address the complexity of public health problems, programs, and policy interventions and to understand the “process of designing and testing solutions” [20].

We drew on the literature on effective research mentoring [12, 13, 22, 23] to examine the mentoring relationship [13, 24]. Research mentorship was defined as: “…a complex and multidimensional process through which emerging scientists acquire the norms and standards, values and attitudes, and knowledge, skills and behaviors to develop into successful independent researchers” [24]. The literature on training in public health research [5, 11, 25–28] was also used to examine learning processes that foster skills development in PHIR.

## Results

### The role of internships and mentorship

In interviews and in semiannual reports, a majority of trainees and mentors stated that embedding students in PHOs had been positive, which reflects the results of the online survey [19]. First and foremost, they reported that the placements provided a unique opportunity for trainees to acquire an understanding of the role and functions of the public health system and to connect with the reality of conducting public health research “on the ground.”

According to exit interviews, the placements enabled trainees to interact with public health practitioners and to understand PHIs. Embedding of trainees in PHOs created the opportunity to experience the development, implementation, and evaluation of interventions: “I was able to reflect and question public health interventions thanks to my internship and my mentor's team that showed me what effective interventions are and how to evaluate them” (EI-17). Likewise, trainees and mentors in telephone interviews reported: “the possibility of experiencing public health” ... “to know the conditions for the implementation of the action” (TI-T8), “to understand priorities” (TI-T6). Trainees gained an understanding of patterns of interactions and relationships within PHO: “My internship allowed me to better understand the situation on the ground, the necessary conditions for innovations, coalitions and changes” (SAER-16).

The placements allowed trainees to appreciate the range of research activities in PHOs and the finality of PHIR: “I saw what can be done in population health research” (EI-27). It also permitted trainees to identify the multidimensionality (e.g., contextual, ethical dimensions) of public health research problems and understand what research questions are of interest to practitioners, according to interviews with mentors. Trainees acquired a knowledge of the processes involved in PHIR and how research informed programs: “I gained a deeper understanding of the structure of public health research, how the evidence is constructed, how decisions are made: all this was new to me” (EI-25). Trainees learned to get closer to decision-makers and to invite knowledge users on their teams from the research onset. Indeed, in exit and in telephone interviews, trainees characterized placements as thought-provoking and formative experiences in public health research.

Simultaneously, the placement was an opportunity to assess the gap between research and practice, and experience intersectorality, which one trainee defined as “the needed reconciliation of the public health practice and the research practice” (EI-27). By being in the practice environment, trainees discovered that they had to learn how to do research in relation with partners, to develop intersectoral working relationships with practitioners (EI-10), to focus research on a common goal with decision-makers (EI-11).

For trainees, mentorship was key to penetrate the field of public health: “The main quality of mentoring is getting us into the public health environment to conduct our projects” (EI-5). This integration was critical for internship success:

“Insertion into a research team makes it possible to engage in learning about research. Mentoring provided me with access to the senior researchers who helped me with my learning, such as how to coordinate a project, make a budget, acquire methodological knowledge, manage a database, and so on. I had access to several researchers who were open and who stimulated the learning of early career researchers. … I have greatly benefited from the leadership of these researchers and their contacts with productive external members” (EI-35).

A successful mentorship involved a reciprocal commitment between mentor and mentee. Engaged and available mentors, who were skilled in PHIR, respectful of trainees, and who drew upon trainees’ strengths, were perceived as successful. For example, one trainee said:

“Not only were my learning objectives and career goals present in all discussions with my mentor, but also the public health vision and purpose. ... The exchanges contributed to the further elaboration and application of public health concepts in my research work” (EI-11).

An engaged trainee whose project fitted the PHO’s priority was seen as stimulating by mentors. Altogether, a good fit between the mentor and the trainee, immersion of the trainee in the mentor’s research team, and productive mentoring dialogs set the conditions for success.

Mentors helped trainees refine their competencies, develop research methods applied to the local context, and provided opportunities for networking and research partnerships. Beyond the mentoring relationship, trainees noted the importance of the research capacity at the PHO and of the leadership of the mentor.

### Benefits of internships and mentorship

The internships and mentorship allowed trainees to apply research skills in context and integrate learning. Integration of learning about PHIR happened in multiple ways: changes in attitudes, a recognition of the complexity of PHIs, a gradual deepening of PHIR, and the development of cross cutting competencies, as shown below.

The confrontation with the realities of the public health field affected trainees’ attitudes towards research. It was a shock for some trainees: “I changed my research topic” (TI-T8). Internships and mentorship encouraged openness: “The program forced us to open up to the world and take a systemic approach; it permitted to get out the dominant train of thought” (EI-16). Trainees became more critical of their research and asked “what is the use of what we do?” (e.g., EI-16, EI-19). Trainees said that they gained maturity and confidence: “I gained more insights as I progressed through my internships” (EI-34); “I built my capacity to ask questions and discuss public health problems” (EI-17). Trainees alluded to a “process of maturation” (EI-16, EI-35) in the ways of seeing things and looking for solutions to public health challenges. Internships and mentorship strengthened trainees’ perspectives on population health intervention research. They said: “The program brought a refreshing perspective by bringing together researchers and decision-makers; without the Program, I would not have had the potential to influence practice” (TI-T6); “It expanded my knowledge and the range of what I thought I could do” (EI-2); “[It] made me realize the importance of knowledge translation” (EI-27). Trainees spoke of becoming more open to the realities of core public health functions and to the need for research to inform practice and have an impact: “I quickly learned this: to change things, it is not sufficient to have knowledge but you need the know-how to put it in practice” (EI-37); “How can we attract attention of decision-makers, how can we measure change?” (EI-3).

Trainees discovered the complexity of real-world public health problems and interventions. They experienced the influence of context on PHIs, including the power relationships between various stakeholders engaged in PHIs (e.g., practitioners; managers): “Now I see the importance of taking into account the realities on the ground” (EI-11).

Many trainees said that they felt better equipped to understand complexity, health equity, and the need for knowledge translation; they also developed critical thinking skills. Trainees started to ask questions like “so what” and “how and under which conditions” can an intervention be successfully implemented.

“Now I ask myself: “what’s the point?”, “what’s it for?”, “what do we do next?”, “what will the managers who work at the Agency do with this?” Thanks to my internship, I familiarized myself with the milieu and saw how, with my mentor, I could get managers and decision-makers to listen. Now I know that my thesis will serve in practical ways the development of healthy public policies” (EI-19).

Trainees gained research experience: “[The internship] ... allowed me to develop a more complex research plan” (EI-3). This is also reflected in their willingness to revisit their conceptual frameworks and adopt a more transdisciplinary approach to research. In some instances, trainees revised their research protocol to more effectively respond to public health priorities. One trainee made this statement:

“The approach to intervention research was a discovery. This changed the way I look at the research process, and got me to think about research in terms of how it should be led in order to influence intervention and be used by decision-makers. Addressing ethics, considering the complexity of evaluation of interventions, all this knowledge has changed my way of thinking and influenced the choice of the theoretical framework of my thesis. This has influenced my way of seeing my research topic. I now have a more systematic view of innovation” (EI-16).

The mentors corroborated in interviews, and in internships reports, that the internships permitted trainees to reorient their research according to their observations.

Throughout their internships, trainees experienced the challenges of applying the principles of intervention research and developed cross-cutting competencies essential to PHIR such as interdisciplinary and intersectoral communication, partnerships, networking, and knowledge translation. Trainees improved their capacity to interact with researchers and knowledge-user partners from different disciplinary perspectives. They expressed comments such as this one:

“I developed an openness to other disciplinary horizons, learned to adjust my language, to involve various actors to address a particular issue, how to work in partnership. I would not have done this so thoroughly without the 4P” (EI-23).

Mentorship in applied public health settings enabled trainees to learn about processes of knowledge translation, including exchange between researchers and decision-makers, and sharing of knowledge “in an appropriate and effective manner” (SAER-30). Trainees also learned to produce research results useful to public health. Summing up her experience, one trainee said:

“My mentor put me in close contact with the stakeholders at the regional level. ... My ability to produce useful results was enhanced. ... The 4P Program brings legitimacy to the trainee, which translates into an authorization to observe, engage in dialogue with practitioners and key decision makers in public health, be heard and considered. This legitimacy and the quality of the knowledge exchanges are linked, and this shapes things to come, i.e. the translation of knowledge into action. The trainee presence in the public health milieu shaped a reciprocal relationship with the mentor” (EI-5).

A majority of trainees spoke of placements as having broadened their research network and facilitated professional connections with practitioners and decision-makers. Networking and knowledge exchange practices with mentors and knowledge users represented the starting point in learning to engage in collaborative PHIR with members of PHOs.

Overall, former trainees said they acquired competencies not taught in doctoral programs. Field placements acted as catalysts for achieving mastery of the 4P Program core competencies. One trainee put it this way:

“The 4P Program acted as a buffer zone between doctoral studies and the reality of the practical environment. It has shown its full worth in accompanying us in the milieu and by facilitating linkages with this milieu” (EI-26).

Nevertheless, many trainees mentioned they had less than perfect public health internships and mentorship experiences in both exit (about 12 out of 33 trainees since 2009) and telephone interviews (about 7 out of 18; sample of trainees since 2003). For example, trainees said: “my mentor was not available” (EI-19; TI-T3; TI-T14; TI-T16); “It was difficult to establish a good mentoring relationship with my mentor” (EI-19); “I don’t think my mentor understood her role” (EI-27); “My research topic was not a priority for the PHO. I didn’t work with the mentor’s team. As a result, I couldn’t develop my skills in collaborative research as I expected” (EI-13); “My mentor’s team was dynamic but there was no opportunity to exchange with other research teams” (EI-06). In telephone interviews, one trainee explained that her/his internship fell short of her/his expectations for a number of reasons: she/he was not asked by her/his mentor to participate in regular team meetings, there were no real opportunities to develop collaborative projects, and no opportunity to be integrated as a full researcher in a PHO after her/his placement (TI-T3).

Internships and mentorship influenced trainees’ career path. Trainees reported that internships opened doors to employment opportunities outside academia and enabled opportunities for collaboration. However, unfavorable experiences could reorient research careers away from public health. Trainees and mentors alike identified characteristics of successful placements, including the volume of research activity in the PHO, the dynamism of the PHO’s sector of activity to which the trainee was attached, and the PHO focus on intervention research.

Public health managers, for their part, felt that three conditions had to be met for successful internships: (1) integration of the trainee within the organizational framework of the PHO, (2) alignment of the trainee research topic with the PHO’s and the research team priorities, and (3) contributions of trainees to innovative research, practice, and evaluation within the PHO. In their eyes, the contribution of the Program was through the insertion of trainees in cutting-edge research and emerging initiatives in public health.

The mentors, trainees, and public health managers provided advice for how to strengthen the quality and improve the impact of research internships. They advised to improve (1) the fit between the trainee, the mentor, and the PHO (ensure the best alignment of the interests of each, make explicit the aspects of the collaboration, and establish the basis for a successful milieu-trainee relationship); (2) the skill sets of mentors (train mentors in PHIR skills, and support them to fulfill their mentoring role); and (3) the development of PHIR skills (a combination of an increased exposure of trainees to PHIR, a stronger intervention focus in trainees’ research, and a more structured mentoring process focusing on the acquisition of specific PHIR skills).

## Discussion

The consistency of findings across methods and sources of data conveys a clear message on the conditions of success of internships and mentorship for PHIR.

Through prolonged internship and mentorship, 4P trainees explored the functional and relational characteristics of the public health system and developed a working knowledge of complex organizations, including structure, staff, activities, and context. This was seen by the Program designers as a prerequisite for the development of PHIR competencies. This is congruent with the observations of Bachrach and Daley [29] who noted a need for “experience-based training and mentoring in the skills needed to lead and collaborate in interdisciplinary teams” (p. 252). Interdisciplinary collaboration skills were one of three core domains, along with knowledge acquisition and knowledge translation, identified by these authors as critical in developing outstanding interdisciplinary population health scientists [10].

Building on social theory [30, 31], Potvin and colleagues provided conceptual grounds to reflect on PHIs and the role of PHIR in supporting innovation and transformation of practices in the “social-health space” [21, 32]. Bilodeau and Potvin [21] argue that “conceiving PHIs as systems, [which] provides a framework for problematizing relationships between interventions and their contexts, and how such relationships transform them both” (p.2), is strategic since “this leads to examining the connections between the various elements of the intervention and context, the network they form and the evolution of that network” (p.2).

In this vein, PHOs represent important research training laboratories to understand the “social-health space.” Indeed, internships in PHOs and mentorships opened a window on the “sociotechnical network,” a hybrid collection of social actors from various sectors (e.g., public administration, civil society, private sector) that connect with knowledge on activities and services and with resources and constraints from the environment [21]. In this vibrant context, the trainee may (1) observe the dynamic context in which decisions are made and interventions are implemented, (2) witness the interactions between these social actors, and (3) experience interdisciplinary research partnerships.

Becoming part of this network meant access to the “black box of PHIs” [21]. The trainee could link intervention “processes, conditions, context, and effects” [33] (p.14). The mentor acted as an operator that permitted the trainee to observe the inner working of the black box of interventions and learn, for example, how an intervention is decided upon and implemented; how it produces its effects; how actors relate to each other, share information, negotiate, connect, and influence intervention design and implementation; how decision-makers manage changing circumstances; and how their decisions impact intervention outcomes. In sum, the “how” questions are critical to a more comprehensive view of the complexity of PHI [21] and are needed to accelerate the effective implementation of evidence-informed public health practice and decision-making [34]. Immersion in the public health milieu promoted a collaborative research workspace in which trainees interacted with knowledge-user partners and experienced the co-construction of knowledge, including issues of transdisciplinarity and partnerships [35], as well as challenges and opportunities for knowledge translation [36, 37].

Consistent with twenty-first century goals for public health training [28] and adult learning principles [38], trainees who benefited most from their internships were exposed to learning situations that engaged them actively in a complex and changing public health system to develop and apply new knowledge relevant to PHIs. Such applied training appears to be valuable in developing the field of PHIR [39], creating the evidence base for population health, and addressing the demand for consequential science [10, 29, 33, 40].

The internship experience was not the same for all trainees and was directly linked to the quality of mentorship. Observed challenges in mentoring coincided fully with the scientific literature regarding the need for an adequate match between mentor and mentee [24], explicit communication strategies and clarification of expectations [14, 22], and formal training in mentoring practices [12, 14, 22, 24, 41, 42] and skills to implement PHIR [26]. The need to better structure mentorship and objectives around PHIR learning (e.g., to introduce focused discussions around designing and testing the effectiveness of PHIs) was identified as key to program improvements. As per the literature on mentorship [12, 14, 42, 43], mentors should be provided with the tools and resources to initiate discussions as well as guide trainees’ observations and research skills development, and mentors should be evaluated.

Successful mentoring also requires immersion in a facilitative environment [13, 43] with an excellent research capacity [22, 42], and that promotes the integration of contributions from diverse disciplines [10]. There is also a role for institutions to oversee mentorship [24, 42] and to promote collaborations between practice, policy, and research [44].

From the Program’s perspective, challenges to creating successful mentored internships in public health include structuring the learning environment and measuring the efficacy of the mentoring process, sustaining a steady engagement of mentors in the Program, and ensuring comprehensive training in collaborative research [25, 29, 35, 37, 45–47]. More broadly, challenges also include maintaining training program funding and supporting new PHIR researchers who completed the Program. Another issue concerns the interface between public health and university. The difficulties involved in bringing together two entities whose mission, vision of research, and priorities are different require clarification and strengthening of collaborations and partnerships [46].

## Conclusion

This study suggests that our program created a successful learning environment that allowed the opening of the “black box” of PHIs and was conducive to the development of PHIR competencies among PhD and post-doctoral trainees. Public health internships and mentorship prepared trainees to engage in “public health research of consequence” [40]. Indeed, quality internships gave trainees the opportunity to experience the development, implementation, and evaluation of public health programs and policy interventions while quality mentorship was conducive to asking consequential questions like “so what” and “how and under which conditions” an intervention could be successful.

Future research to improve PHIR training include (1) identifying which aspects of mentorship are most conducive to meaningful, long-term integration of researchers in PHOs and to orienting the research career of young investigators towards transdisciplinary collaborative research; (2) understanding the dynamic context in which trainees, mentors, and institutions produce innovative research, practice, and policies; and (3) measuring the conditions in which academia and the public health system can work durably to bridge research and practice in public health.

## Additional file

Additional file 1: Telephone interviews with trainees. Telephone interviews with mentors. Exit interviews - template. Semiannual evaluation reports. Online Survey - trainees. Online Survey - mentors with trainees. (ZIP 999 kb)
