# Supplementary material for: Population health intervention research training: the value of public health internships and mentorship
Source: Public Health Rev. 2018 Apr 2;39:6. doi: 10.1186/s40985-018-0084-9 (PMC5879914; doi:10.1186/s40985-018-0084-9)
Supplement: Supplementary file 1 — Telephone interviews with trainees. Telephone interviews with mentors. Exit interviews - template. Semiannual evaluation reports. Online Survey - trainees. Online Survey - mentors with trainees. (ZIP 999 kb) [file 40985_2018_84_MOESM1_ESM.zip › Qual tel interviews with trainees - template.pdf]

Évaluation du Programme stratégique de formation en recherche transdisciplinaire sur les interventions en santé publique: Promotion, Prévention et Politiques Publiques

Grille d'entrevue téléphonique : Boursiers (14-07-2014)

Introduction

1. Pour commencer, dites-moi pourquoi et comment êtes-vous venu à participer au Programme 4P/Trans?

2. Pourriez-vous me situer en me décrivant votre participation au Programme 4P/Trans en termes de: dates, durée (au sein du Programme; + durée stage), lieux de participation (stage et séminaires) ?

3. Outre la bourse, quels éléments du Programme vous paraissaient les plus importants ou intéressants lorsque vous avez-posé votre candidature?

- Séminaires
- Mentorat
- Stage en milieu de santé publique

Nous reviendrons plus loin sur ces aspects (Q10)

Parcours

2. Depuis que votre implication dans le Programme 4P/Trans est terminée, quels ont été vos postes et activités professionnelles? Et quel est votre statut actuel?

pour ceux ayant un double rattachement (université / santé publique) : comment décririez-vous votre niveau d'implication ou d'investissement dans ces deux milieux? Quels sont les avantages et désavantages d'un rattachement double pour vous?

3. Quels objectifs de carrière poursuivez-vous?
- lieu, employeur
- type d'activités
- nature des recherches (s'il y a lieu)

Vos objectifs ont-ils changé en cours de participation ou depuis votre participation au Programme 4P/Trans?

4. Idéalement, comment voyez-vous votre progression de carrière à court terme, et à plus long terme (5 à 10 ans)

Pratiques actuelles

5. Dans vos activités professionnelles actuelles (recherche et/ou autres), appliquez-vous des éléments (contenus, habilités, attitudes, contacts) acquis ou appris dans le cadre du Programme 4P/Trans?

Si oui, lesquels, et comment?

6. Pouvez-vous donner un exemple concret de l'influence du Programme 4P dans vos pratiques actuelles?

Par rapport aux aspects suivants :

- Accent sur une vision large et transdisciplinaire de problématiques de santé des populations
- Jusqu'à quel point intégrez-vous une diversité de disciplines dans vos travaux actuels?
    - Considérez-vous qu'il y a eu une certaine évolution dans votre façon de faire la recherche ou une certaine adaptation de vos méthodes depuis votre formation disciplinaire d'origine?
    - Comment avez-vous composé avec votre formation disciplinaire d'origine lors de votre participation au Programme?
- Dimensions éthiques de la recherche interventionnelle
    - Avec qui avez-vous échangé sur ces dimensions dans le cadre de votre participation? Sur quels aspects (vis-à-vis des CER, enjeux d'intervention, posture du chercheur)? Comment ces échanges ont-ils influencé vos pratiques actuelles?
- Travail en partenariat
    - qui sont vos partenaires?
- Quels sont vos liens avec les décideurs et autres utilisateurs des résultats de recherche
- Partage-transfert des connaissances, application des connaissances

7. Faites-vous de la recherche actuellement ? Quelle est sa nature/ses objets/populations? S'agit-il de la recherche interventionnelle? Et si oui, comment ceci se traduit-il : par la recherche directement sur l'intervention ou la recherche orientée vers l'intervention? Pourquoi?

8. Travaillez-vous actuellement avec des individus ou des organisations avec qui vous avez collaboré ou que vous avez connus dans le Programme 4P/Trans? Comment ces collaborations ont pris naissance? Auraient-elles existé si vous n'aviez pas participé au Programme 4P/Trans?

Contribution du Programme

9. De façon globale, quelle est, ou a été, la contribution du Programme 4P/Trans à vos pratiques de recherche/professionnelles actuelles? Comment vos pratiques seraient différentes si vous n'aviez pas participé au Programme 4P/Trans?

Observez- vous des différences dans vos pratiques par rapport à celles de vos confrères étudiants qui ne faisaient pas partie du Programme 4P?

10. Pour vous, quelles sont la nature et l'utilité de la contribution des composantes du Programme 4P/Trans à vos pratiques, vos orientations de recherche ou de pratique actuelles?

- séminaires: la nature et l'utilité de la contribution
    - Plus spécifiquement : contribution pour le partage des connaissances? Pour la conduite des études en partenariat?
    - Plus spécifiquement : comment évaluez-vous les façons dont la transdisciplinarité a été abordée lors des séminaires?
- mentorat reçu dans le cadre du Programme: la nature et l'utilité de la contribution
    - Ce mentorat se poursuit-il actuellement?
- insertion en milieu de santé publique: la nature et l'ampleur de la contribution.
    - Quelle était l'intensité (durée, nombre d'activités et fréquence par mois, degré d'intégration aux équipes, productions communes) de votre expérience d'insertion?
    - Comment cette intensité a-t-elle affecté ce qui vous avez retenu ou acquis de l'expérience?

11. Est-ce que votre participation a engendré d'autres effets pour vous? Si oui, lesquels?

12. Pour vous, comment le Programme a-t-il contribué à vos perspectives de carrière?

13. Comment les connaissances, habiletés et attitudes développées au cours du Programme permettent-elles de répondre aux besoins changeants et émergents en santé publique?

Programme idéal

14. Quels sont les facteurs facilitants à la formation des étudiants en recherche interventionnelle sur la santé des populations? … qui font en sorte que les jeunes chercheurs choisissent de travailler sur les interventions axées sur les besoins des milieux de santé publique?

Quels sont les barrières à la formation en recherche interventionnelle sur la santé des populations?

15. Prenant en compte les objectifs du Programme ainsi que les besoins des étudiants et des milieux de santé publique, pourriez-vous me décrire un programme de formation « idéal » en recherche interventionnelle en santé des populations?

16. Quelles recommandations pourriez-vous formuler qui feraient en sorte que le Programme soit amélioré et renforcé et qu'il réponde encore mieux aux besoins?
