# Supplementary material for: Population health intervention research training: the value of public health internships and mentorship
Source: Public Health Rev. 2018 Apr 2;39:6. doi: 10.1186/s40985-018-0084-9 (PMC5879914; doi:10.1186/s40985-018-0084-9)
Supplement: Supplementary file 1 — Telephone interviews with trainees. Telephone interviews with mentors. Exit interviews - template. Semiannual evaluation reports. Online Survey - trainees. Online Survey - mentors with trainees. (ZIP 999 kb) [file 40985_2018_84_MOESM1_ESM.zip › Qual tel interviews with mentors - template.pdf]

Évaluation du Programme stratégique de formation en recherche transdisciplinaire sur les interventions en santé publique: Promotion, Prévention et Politiques Publiques (4P)

Grille d'entrevue téléphonique : Mentors (14-07-2014)

Introduction

1. Pour commencer, dites-moi pourquoi et comment êtes-vous venu à participer au Programme 4P?

2. Pourriez-vous me situer en me décrivant votre participation au Programme 4P en termes de : dates, durée, formes de participation?

Besoins

3. Selon vous, quels besoins le Programme 4P vise-t-il à combler chez les personnes en formation de recherche en santé publique? Chez les milieux de santé publique? Quelle est l'ampleur ou l'importance de ces besoins – quel est le problème que le Programme tente de résoudre selon vous?

4. À quel point le Programme 4P est-il adapté aux besoins des milieux de santé publique? En quoi est-il adapté, et en quoi ne l'est-il pas?

5. Le Programme arrive-t-il à répondre aux besoins des milieux? Pourriez-vous donner des exemples, soit positifs ou négatifs de cette réponse aux besoins?

6. Plus particulièrement, est-ce que le Programme apporte une aide à la solution de problèmes complexes de santé publique? Si oui, comment? Si non, pourquoi pas?

Pratiques actuelles

7. À quel point et comment les aspects suivants du Programme influencent-ils vos pratiques actuelles (en comparant avant et après l'accueil du boursier)?

- Accent sur une vision large et transdisciplinaire de problématiques de santé populationnelle
- Dimensions éthiques de la recherche interventionnelle
- Travail en partenariat

- Liens avec les décideurs et autres utilisateurs des résultats de recherche
- Partage-transfert des connaissances, application des connaissances

8. Travaillez-vous actuellement avec des individus ou des organisations avec qui vous avez collaboré ou que vous avez connus dans le Programme ? Comment, et pourquoi, ou pourquoi pas? Ces collaborations auraient-elles existé si vous n'aviez pas participé au Programme 4P/Trans?

Contribution du Programme

9. À quel point, selon vous, les boursiers ont-ils évolué dans leur compréhension et leurs capacités en recherche interventionnelle en santé des populations, grâce au Programme?

Dans quels domaines leur évolution était-elle le plus et le moins marquée?

10. Quelle était la nature et l'utilité de la contribution des composantes du Programme 4P à la progression des boursiers dans l'acquisition des compétences visées par le programme? :

- bourses?
- séminaires?
- mentorat ?
- insertion en milieu de santé publique ?

11. De votre point de vue, quelles étaient les contributions les plus importantes du Programme au développement des capacités :

- des boursiers à entreprendre une carrière de recherche interventionnelle?
- des milieux de santé publique à réponse aux besoins changeants et émergents en santé publique?

12. Le boursier a-t-il fait une différence, changé quelque chose dans votre milieu? Si oui, laquelle? Est-ce majeure ou mineure? Durable ou temporaire?

13. Est-ce que vous ou votre organisation avez offert au boursier des opportunités professionnelles/de carrière suite au stage? Lesquelles? Pourquoi, ou pourquoi pas?

Programme idéal

14. Quels sont les facteurs facilitants à former des étudiants en recherche interventionnelle en santé des populations? À faire en sorte que les jeunes chercheurs choisissent de travailler sur les interventions axées sur les besoins en santé publique?

Quelles sont les barrières à la formation en recherche en santé des populations?

15. Prenant en compte les objectifs du Programme ainsi que les besoins des étudiants et des milieux de santé publique, quelles recommandations pourriez-vous formuler qui feraient en sorte que le Programme réponde encore mieux aux besoins?
