# Supplementary material for: Population health intervention research training: the value of public health internships and mentorship
Source: Public Health Rev. 2018 Apr 2;39:6. doi: 10.1186/s40985-018-0084-9 (PMC5879914; doi:10.1186/s40985-018-0084-9)
Supplement: Supplementary file 1 — Telephone interviews with trainees. Telephone interviews with mentors. Exit interviews - template. Semiannual evaluation reports. Online Survey - trainees. Online Survey - mentors with trainees. (ZIP 999 kb) [file 40985_2018_84_MOESM1_ESM.zip › Online Survey - trainees.pdf]

Évaluation du Programme stratégique de formation en recherche transdisciplinaire sur les interventions en santé publique: Promotion, Prévention et Politiques Publiques

Sondage auprès des boursiers (11-02-2014)

Merci de bien vouloir participer à ce sondage qui vise à connaître votre point de vue sur le Programme de formation 4P. Dans ce document, le genre masculin est utilisé comme générique dans le seul but de ne pas alourdir le texte.

Veuillez compléter le sondage d'ici le **3 mars 2014**. Le questionnaire comprend quatre sections (Intérêt pour le Programme; Expérience au sein du Programme; Après le Programme; Profil du boursier) et dure environ **20 minutes**. Au besoin, il vous est possible de remplir le questionnaire en plusieurs sessions. Lorsque vous quittez le document, vos informations sont enregistrées et vous pouvez le reprendre au moment qui vous convient.

Tous les renseignements fournis sont strictement confidentiels. Seul l'assistant de recherche engagé pour le traitement des données aura accès aux données identifiables. Lui-même signera un engagement à la confidentialité des données. Pour toute question, n'hésitez pas à communiquer avec madame Anne-Marie Hamelin, directrice-adjointe, Programme de formation 4P, par téléphone : 514 398 3231 ou par courriel : 4P-amh.eboh@mcgill.ca.

Avant de procéder, veuillez attester de votre consentement, s'il vous plaît.

Consentement

J'ai lu les renseignements sur ce sondage. J'ai eu l'occasion de me renseigner sur ma participation et d'obtenir les informations nécessaires pour le compléter. Je comprends que tous les renseignements que je fournirai seront strictement confidentiels et que seul l'assistant de recherche engagé pour le traitement des données aura accès aux données identifiables.

En remplissant ce questionnaire, je consens à participer à ce sondage.

- ○ J'accepte
- ○ Je n'accepte pas

Évaluation du Programme stratégique de formation en recherche transdisciplinaire sur les interventions en santé publique: Promotion, Prévention et Politiques Publiques

Sondage auprès des boursiers (11-02-2014)

Section 1: Intérêt pour le Programme

1. Comment avez-vous entendu parler du Programme?

○ Par mon directeur de thèse ou superviseur postdoctoral
○ Par un autre professeur
○ Par un autre étudiant
○ Par le site web
○ Autre (spécifier) ______________________

2. Quels aspects du Programme vous ont intéressé au moment de poser votre candidature?

Cocher tous ceux qui s'appliquent.

☐ Bourse
☐ Séminaires
☐ Stage en milieu de santé publique
☐ Mentorat
☐ Vision large et transdisciplinaire
☐ Réseau
☐ Autre (spécifier) ______________________

3. À quel point la bourse du Programme était-elle importante dans votre décision de poser votre candidature?

○ Essentielle - je n'aurais pas posé ma candidature
○ Importante - un facteur clé mais pas le seul
○ Marginale - un facteur mais pas le plus important
○ Pas importante du tout

Section 2: Expérience au sein du Programme

Nous cherchons à savoir à quel point votre expérience au Programme a contribué à chacun des résultats attendus. Pour chaque élément où il y aura eu contribution, nous vous demanderons à quel point chacun des éléments du Programme (par exemple, le mentorat) y a contribué.

4. À quel point votre participation au Programme a influencé votre sujet de recherche doctoral ou postdoctoral?

○ Pas du tout
○ Un peu
○ Assez
○ Beaucoup
○ Énormément

PROGRAMME DE FORMATION
TRAINING PROGRAM
PROMOTION, PRÉVENTION ET POLITIQUES PUBLIQUES
PROMOTION, PREVENTION AND PUBLIC POLICY

Évaluation du Programme stratégique de formation en recherche transdisciplinaire sur les interventions en santé publique: Promotion, Prévention et Politiques Publiques

Sondage auprès des boursiers (11-02-2014)

5. À quel point votre participation au Programme vous a permis, dans le cadre de vos travaux de 3ième cycle, ...

Répondre aux 8 questions suivantes:

	Pas du tout	Un peu	Assez	Beaucoup	Énormément
De développer une vision plus large des problématiques d'intervention de santé publique?	○	○	○	○	○
De développer une ouverture vers d'autres disciplines?	○	○	○	○	○
De développer des habiletés à échanger avec des collègues d'autres domaines?	○	○	○	○	○
De développer des habiletés à travailler avec des gens d'autres domaines?	○	○	○	○	○
D'accroître vos habiletés à mener des études en partenariat avec différents groupes d'intérêt? (Si vous ne travaillez pas avec des groupes d'intérêt, cochez "Pas du tout")	○	○	○	○	○
De développer votre compréhension des dimensions éthiques de la recherche appliquée en santé des populations?	○	○	○	○	○
De vous rendre plus apte à partager vos connaissances et vos résultats de recherche avec les communautés d'intérêt?	○	○	○	○	○
De vous rendre plus apte à partager vos connaissance et vos résultats de recherche avec des décideurs?	○	○	○	○	○

6. À quel point votre insertion en milieu de santé publique, incluant le mentorat de santé publique, a contribué:

Répondre aux 8 questions suivantes:

	Pas du tout	Un peu	Assez	Beaucoup	Énormément
À l'élargissement de votre vision des problématiques d'intervention en santé publique?	○	○	○	○	○
Au développement d'une ouverture vers d'autres disciplines?	○	○	○	○	○
À l'accroissement de vos habiletés à échanger avec des collègues d'autres domaines?	○	○	○	○	○
À l'accroissement de vos habiletés à travailler avec des gens d'autres domaines?	○	○	○	○	○
À l'accroissement de vos habiletés à mener des études en partenariat avec différents groupes d'intérêt? (Si vous ne travaillez pas avec des groupes d'intérêt, cochez "Pas du tout")	○	○	○	○	○
Au développement de votre compréhension des dimensions éthiques de la recherche appliquée en santé des populations?	○	○	○	○	○
À vous rendre plus apte à partager vos connaissances et vos résultats de recherche avec les communautés d'intérêt?	○	○	○	○	○
De vous rendre plus apte à partager vos connaissances et vos résultats de recherche avec des décideurs?	○	○	○	○	○

7. À quel point l'ensemble des séminaires du Programme a contribué:
Répondre aux 8 questions suivantes:

	Pas du tout	Un peu	Assez	Beaucoup	Énormément
À l'élargissement de votre vision des problématiques d'intervention en santé publique?	○	○	○	○	○

Au développement d'une ouverture vers d'autres disciplines?	○	○	○	○	○
À l'accroissement de vos habiletés à échanger avec des collègues d'autres domaines?	○	○	○	○	○
À l'accroissement de vos habiletés à travailler avec des gens d'autres domaines?	○	○	○	○	○
À l'accroissement de vos habiletés à mener des études en partenariat avec différents groupes d'intérêt? (Si vous ne travaillez pas avec des groupes d'intérêt, cochez "Pas du tout")	○	○	○	○	○
Au développement de votre compréhension des dimensions éthiques de la recherche appliquée en santé des populations?	○	○	○	○	○
À vous rendre plus apte à partager vos connaissances et vos résultats de recherche avec les communautés d'intérêt?	○	○	○	○	○
De vous rendre plus apte à partager vos connaissance et vos résultats de recherche avec des décideurs?	○	○	○	○	○

8. Au cours du Programme, avez-vous développé des partenariats avec des organismes avec lesquels vous n'aviez jamais collaboré auparavant?

○ Oui
○ Non

9. Pouvez-vous donner un exemple de partenariat que vous avez développé?

10. Est-ce que vous trouvez que le Programme est adapté aux besoins des milieux de santé publique (ou des milieux où les résultats de recherche en santé des populations peuvent être appliqués)?

○ Oui
○ Partiellement
○ Non

11. Pourquoi?

[ ]

12. À quel point avez-vous trouvé que c'était facile ou difficile d'équilibrer les intérêts de votre milieu de santé publique, les intérêts du Programme et vos intérêts à terminer vos études doctorales ou postdoctorales?

- ○ Très difficile
- ○ Difficile
- ○ Parfois facile, parfois difficile
- ○ Facile
- ○ Très facile

Pouvez-vous nous dire ce qui fait que cet équilibre était très difficile, difficile, ou parfois facile, parfois difficile?

[ ]

13. Considérez-vous que votre participation au Programme engendre (ou a engendré) des risques pour vous et votre doctorat/stage postdoctoral?

- ○ Oui
- ○ Non

A-t-elle engendré des risques en termes de:

Cocher tous ceux qui s'appliquent.

- ☐ Sollicitations pour entreprendre des travaux ou des projets additionnels
- ☐ Prolongement de votre programme/délai d'obtention du diplôme
- ☐ Abandon du Programme
- ☐ L'indépendance de votre démarche intellectuelle ou scientifique
- ☐ La rigueur de votre processus de recherche
- ☐ Tension entre mentor du Programme et directeur de thèse
- ☐ Autres _______________________

Comment avez-vous composé avec ces risques?

[ ]

Section 3: Après le Programme

14. Dans vos travaux actuels, à quel point:

Choisir l'énoncé le plus pertinent.

Évaluation du Programme stratégique de formation en recherche transdisciplinaire sur les interventions en santé publique: Promotion, Prévention et Politiques Publiques

Sondage auprès des boursiers (11-02-2014)

	Pas du tout	Un peu	Assez	Beaucoup	Énormément
Utilisez-vous les connaissances, les habiletés et les attitudes que vous avez développées au cours du Programme?	○	○	○	○	○
Êtes-vous en lien avec le réseau que vous avez développé au cours du Programme?	○	○	○	○	○
Sentez-vous que le Programme a augmenté votre capacité à contribuer à la solution des problèmes complexes de santé publique?	○	○	○	○	○
Adoptez-vous une vision large et transdicisplinaire?	○	○	○	○	○
Travaillez-vous en association avec différents groupes d'intérêt (comme un organisme professionnel, une municipalité, un regroupement de chercheurs, des organismes communautaires, une association d'Autochtones, un comité de citoyens)?	○	○	○	○	○
Prenez-vous en compte les dimensions éthiques de la recherche appliquée?	○	○	○	○	○
Partagez-vous vos connaissances et vos résultats de recherche avec les communautés d'intérêt?	○	○	○	○	○
Partagez-vous vos connaissances et vos résultats de recherche avec des décideurs? (Si vous ne travaillez pas avec des décideurs, cochez "Pas du tout")	○	○	○	○	○
Intégrez-vous une approche participative (i.e. une approche qui fait appel à la participation active des membres de la communauté à	○	○	○	○	○

toutes les étapes de la recherche, dont l'origine de la question de recherche vient des préoccupations de la communauté, et dont la nature des résultats de recherche sera pertinente et utilisable par la communauté d'intérêt)?

Section 4: Profil du boursier

15. Quel est votre sexe?

- ○ Femme
- ○ Homme
- ○ Autre ______________________

16. Quel est le plus haut niveau d'éducation que vous ayez atteint (diplôme obtenu)?

Cocher plus d'une case au besoin

- ☐ Maîtrise
- ☐ Ph.D.
- ☐ Doctorat en médecine
- ☐ Autre ______________________

17. À quelles institutions êtes-vous rattaché?

Identifier toutes les institutions d'appartenance et indiquer à quel titre vous êtes rattaché.
Cocher la boîte vis-à-vis votre institution principale.

	Institution principale	Titre
ASPC	☐	
MSSS	☐	
INSPQ	☐	
DSP de Montréal	☐	
DSP de la Vieille Capitale	☐	

DSP de la Montérégie	☐	
DSP de l'Estrie	☐	
Régie régionale de la santé et des services sociaux du Nunavik	☐	
Autre DSP (spécifier)	☐	
CSSS de la Vieille Capitale	☐	
CSSS Bordeaux-Cartierville	☐	
CSSS de la Montagne	☐	
CSSS Jeanne-Mance	☐	
Autre CSSS (spécifier)	☐	
CLSC (spécifier)	☐	
Université McGill	☐	
Université de Montréal	☐	
Université de Sherbrooke	☐	
Université Laval	☐	
Université du Québec à Montréal	☐	
Institut National de la Recherche Scientifique	☐	
École Nationale d'Administration Publique	☐	
Université d'Ottawa	☐	

Autre université (spécifier)	☐	
Autre institution (spécifier)	☐	

18. Quel est votre statut présentement?

Choisir tous ceux qui s'appliquent.

- ☐ Étudiant doctoral
- ☐ Stagiaire postdoctoral
- ☐ Chercheur - universitaire
- ☐ Chercheur - autre type d'institution publique
- ☐ Chercheur - secteur privé
- ☐ Agent de planification, de programmation et de recherche
- ☐ Professeur
- ☐ Médecin en clinique
- ☐ Médecin en santé publique
- ☐ Conseiller scientifique
- ☐ Consultant
- ☐ Chargé de cours
- ☐ Autre ________________________

19. Quel est votre domaine de recherche?

Choisir l'énoncé le plus pertinent.

- ○ Promotion de la santé (incl. inégalités sociales de santé)
- ○ Prévention
- ○ Politiques publiques/organisation des services de santé
- ○ Recherche fondamentale/épidémiologie/modélisation
- ○ Éthique
- ○ Autre ________________________

20. Généralement, quel est le principal type de méthode utilisé dans vos projets de recherche?

Choisir l'énoncé le plus pertinent

- ○ Méthode qualitative
- ○ Méthode quantitative
- ○ Méthodologie mixte

21. Diriez-vous que l'objet principal de vos travaux actuels...

Choisir l'énoncé le plus pertinent.

- ○ Porte directement sur des interventions (i.e. développement; évaluation; mesure d'impact) pertinentes à la santé publique

- ○ Ne porte pas directement sur une intervention, mais est orienté vers l'intervention pertinente à la santé publique (e.g., une étude visant le changement de pratiques) et dont les résultats serviront à moyen terme à développer ou à améliorer une intervention
- ○ Ne concerne pas l'intervention, mais est d'intérêt pour la santé publique
- ○ Autre ______________________

22. Est-ce que vous avez reçu des <u>subventions</u> d'organismes nationaux ou provinciaux au cours des 5 dernières années?
Choisir tout ce qui s'applique et indiquer le nom de l'organisme (ou des organismes).

- ☐ Oui, nationaux ______________________
- ☐ Oui, provinciaux ______________________
- ☐ Non

23. Est-ce que vous avez reçu des <u>bourses</u> d'organismes nationaux ou provinciaux au cours des 5 dernières années?
Choisir tout ce qui s'applique et indiquer le nom de l'organisme (ou des organismes).

- ☐ Oui, nationaux ______________________
- ☐ Oui, provinciaux ______________________
- ☐ Non

Avez-vous d'autres commentaires sur le Programme et votre participation?
