# Supplementary material for: Population health intervention research training: the value of public health internships and mentorship
Source: Public Health Rev. 2018 Apr 2;39:6. doi: 10.1186/s40985-018-0084-9 (PMC5879914; doi:10.1186/s40985-018-0084-9)
Supplement: Supplementary file 1 — Telephone interviews with trainees. Telephone interviews with mentors. Exit interviews - template. Semiannual evaluation reports. Online Survey - trainees. Online Survey - mentors with trainees. (ZIP 999 kb) [file 40985_2018_84_MOESM1_ESM.zip › Exit interviews - template.pdf]

Programme stratégique de formation en recherche transdisciplinaire sur les interventions en santé publique: Promotion, Prévention et Politiques Publiques

Grille d'entrevue de fin de Programme

Date : ....

Nom ……... Niveau : □ Doctorat; □ Post-doctorat

Date d'entrée dans le Programme ....

Dates de congés/prolongation (le cas échéant) …………………

Comment ça va?

Où en êtes-vous dans votre cheminement académique? (où en êtes-vous dans la rédaction doctorale)

Est-ce que votre objet de recherche a changé? Si oui, comment?

1. Quelles sont les connaissances et habiletés spécifiques acquises au cours du Programme depuis votre entrée? (Qu'est-ce que vous avez retenu? Qu'est ce qui a été le plus utile pour vous personnellement et dans votre rôle professionnel?)

2. Avez-vous atteint les objectifs que vous vous étiez fixés? Et dans le temps voulu?

3. Y a-t-il des connaissances et habiletés spécifiques que vous auriez aimé acquérir mais que vous n'avez pu acquérir?

4. Comment ces connaissances et habiletés vous aideraient à mieux atteindre vos objectifs de carrière?

5. Est-ce que l'importance accordée aux compétences que vous vouliez acquérir est encore le même aujourd'hui?

6. Parlez-nous des forces et des faiblesses du Programme.

7. Plus spécifiquement, dites-nous ce que vous pensez des composantes du Programme:

    7.1 Insertion en milieu de SP

        Plan personnalisé

        Stratégie de mentorat

        Évaluations semestrielles

    7.2 Programme complémentaire

        Contenu

        Sert-il l'acquisition de compétences transversales? (vision large et transdiciplinaire, partenariat avec divers groupes d'intérêt et autres chercheurs?)

        Favorise-t-il une plus grande application des résultats de recherche du boursier à l'amélioration de la santé des populations? Comment?

        Parvient-il à renforcer chez le boursier la perspective de réduction des inégalités sociales de santé? Habilite-t-il le boursier à mieux contribuer à la réduction des inégalités sociales de santé?

8. Quelles sont les forces personnelles que vous avez utilisées et qui ont contribué à votre réussite?

9. D'autres idées pour le Programme ? Vos recommandations?
